# Unraveling the Role of Perovskite in Buried Interface Passivation

**DOI:** 10.1021/acsami.3c13085

**Published:** 2023-11-22

**Authors:** Chittaranjan Das, Rajarshi Roy, Mayank Kedia, Małgorzata Kot, Weiwei Zuo, Roberto Félix, Tomasz Sobol, Jan Ingo Flege, Michael Saliba

**Affiliations:** ‡ Institute for Photovoltaics (*ipv*), University of Stuttgart, Pfaffenwaldring 47, 70569 Stuttgart, Germany; § Helmholtz Young Investigator Group, IEK5-Photovoltaik, Forschungszentrum Jülich, 52425 Jülich, Germany; ∥ Chair of Applied Physics and Semiconductor Spectroscopy, Brandenburg University of Technology Cottbus-Senftenberg, Konrad-Zuse-Straße 1, 03046 Cottbus, Germany; ⊥ Department Interface Design, Helmholtz-Zentrum Berlin für Materialien und Energie GmbH (HZB), Hahn-Meitner-Platz 1, 14109 Berlin, Germany; # SOLARIS National Synchrotron Radiation Centre, Jagiellonian University, 31-007 Krakow, Poland

**Keywords:** perovskite solar cells, interface, defects, photoemission spectroscopy

## Abstract

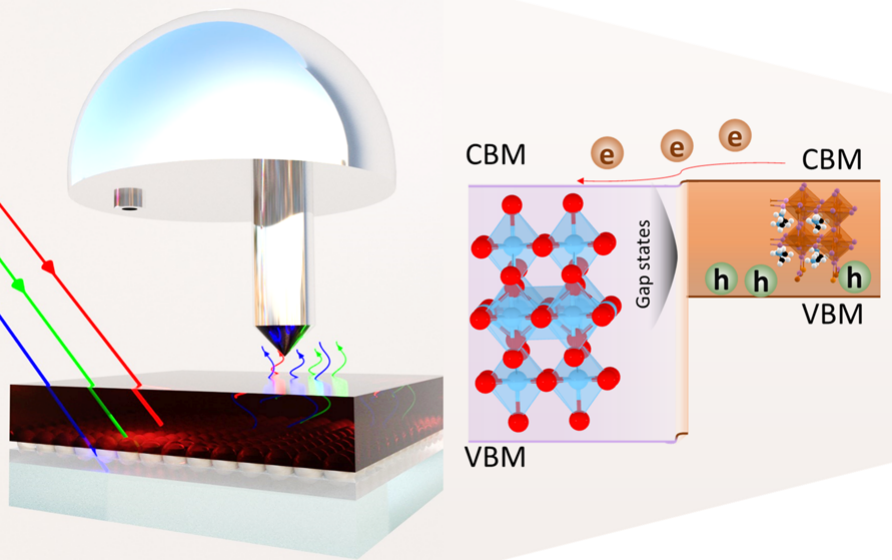

Interfaces in perovskite solar cells play a crucial role in their overall performance, and therefore, detailed fundamental studies are needed for a better understanding. In the case of the classical n–i–p architecture, TiO_2_ is one of the most used electron-selective layers and can induce chemical reactions that influence the performance of the overall device stack. The interfacial properties at the TiO_2_/perovskite interface are often neglected, owing to the difficulty in accessing this interface. Here, we use X-rays of variable energies to study the interface of (compact and mesoporous) TiO_2_/perovskite in such a n–i–p architecture. The X-ray photoelectron spectroscopy and X-ray absorption spectroscopy methods show that the defect states present in the TiO_2_ layer are passivated by a chemical interaction of the perovskite precursor solution during the formation of the perovskite layer and form an organic layer at the interface. Such passivation of intrinsic defects in TiO_2_ removes charge recombination centers and shifts the bands upward. Therefore, interface defect passivation by oxidation of Ti^3+^ states, the organic cation layer, and an upward band bending at the TiO_2_/perovskite interface explain the origin of an improved electron extraction and hole-blocking nature of TiO_2_ in the n–i–p perovskite solar cells.

## Introduction

Perovskite solar cells (PSCs) have made remarkable progress in power conversion efficiency (PCE), increasing from 3.8% in 2009 to an impressive 26.1% in 2023.^1,2^ Initially, research efforts were predominantly centered on perfecting the perovskite absorber layer, with a focus on achieving low defect density, high crystallinity, large grain size, optimal optical properties, and proper band alignment with the respective charge extraction layers.^3−5^ Subsequently, the spotlight shifted toward enhancing the perovskite film through surface modifications^4,6−9^ and optimizing the interfaces between the perovskite layer and charge-transporting materials.^7,10−17^ This shift in focus was driven by the recognition that these factors are critical in the quest for highly efficient solar cells.

In the pursuit of enhancing the PSC performance through interface optimization, researchers have dedicated their efforts to refining the interface between metal oxides and perovskite materials.^18,19^ For instance, the widely employed hole-blocking layer (HBL), particularly TiO_2_, contains oxygen vacancies that effectively transform this wide-bandgap material into a semiconductor.^20,21^ Oxygen vacancies in the TiO_2_ film induce defect states, leading to the trap of photogenerated charges at the TiO_2_–perovskite interface, thereby impacting both the efficiency and stability of PSCs.^22^ To circumvent the effect of the defect states of TiO_2_ at the interface of HBL/perovskite, the surface of TiO_2_ has been modified with various organic and inorganic layers, such as C_60_, NaS, sulfur, and fluorine-based compounds.^23−29^ Therefore, delving into the interfacial properties of PSCs will enhance our understanding of interface modifications for improved device performance.

Among numerous techniques, photoelectron spectroscopy (PES) has been widely employed for the in-depth analysis of electronic device interfaces. PES directly identifies chemical reactions occurring at material surfaces and interfaces when interfacing with another layer, enabling a comprehensive understanding of their electronic properties and, consequently, their band alignment.^30−33^ For instance, in the context of perovskite film interfaces on various substrates, Olthof et al. observed a strong correlation between the initial perovskite growth and the surface chemistry of the substrate.^34^ This observation suggests that changes in the interfacial band edge position have a significant effect on the performance of PSCs. In a related study by Shallcross et al., the interface between TiO_2_ and the perovskite film was investigated through sequential deposition, revealing that the surface of the TiO_2_ film exhibits a high density of defect states that directly influence the chemistry of the subsequently grown perovskite film.^35^ The combination of the defective interface and non-perovskite chemistry of the perovskite film at the interface causes a lower charge separation. However, in the literature, the PCE values of TiO_2_/perovskite-based n–i–p solar cells show high performances, around 20%,^36^ suggesting that this interface may have fewer challenges compared to the NiO_*x*_/perovskite interface in p–i–n architectures.^37^

It is worth noting that the studies conducted on TiO_2_ in combination with vapor-deposited perovskite interfaces should not be directly extrapolated to infer the properties of TiO_2_ interfacing with spin-coated perovskite films. We hypothesize that the process of spin-coating perovskite films onto the TiO_2_ layer, involving solution-based deposition, significantly influences the chemical and electronic characteristics of the TiO_2_/perovskite interface. This differs substantially from non-solution processes, such as vapor deposition. Therefore, in the subsequent discussion, when we refer to perovskites, we specifically mean those produced through solution-based methods, like spin coating.

In this work, we studied the chemical and electronic properties of the TiO_2_/triple cation perovskite with a 1.62 eV bandgap^38^ interface using X-ray photoelectron spectroscopy (PES) methods. In our study, we utilized diluted perovskite solutions with concentrations of 0.2, 0.5, and 1.2 M, yielding approximately 5, 15, and 400 nm thicknesses. These solutions were spin-coated onto a TiO_2_ film, and we employed surface-sensitive soft X-ray photoelectron spectroscopy (SOXPES) and bulk-sensitive hard X-ray photoelectron spectroscopy (HAXPES) techniques for investigation. Our results showed that the bare TiO_2_ film had in-gap states, but these were effectively reduced during perovskite spin coating. At the TiO_2_/perovskite interface, we observed organic cations, metallic Pb^0^ states, and beneficial upward band bending on the TiO_2_ side. These findings suggest defect passivation and advantageous band bending as a result of the perovskite precursor interaction with the TiO_2_ layer, which likely contributes to the high efficiency of TiO_2_/perovskite-based PSCs.

## Results and Discussion

**Figure 1:**
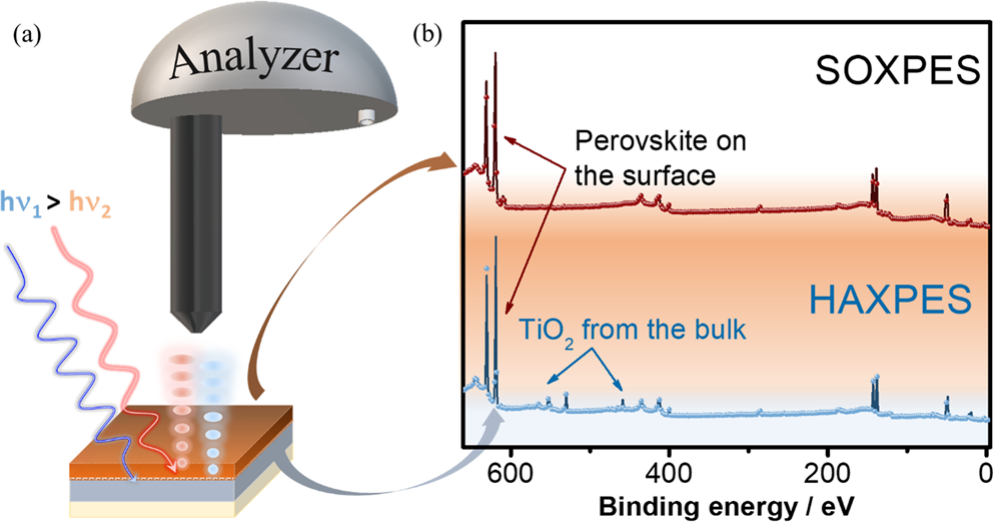
(a) Schematic diagram of the SOXPES and HAXPES measurement setup and (b) survey spectra collected with SOXPES and HAXPES on TiO_2_/perovskite.

To study the interface of the TiO_2_/perovskite, we use a soft X-ray source at an 880 eV energy and a hard X-ray source at a 2.0 keV energy on different thicknesses of perovskite deposited onto TiO_2_. A schematic diagram of the depth-selective SOXPES and HAXPES methods is shown in Figure 1a. Figure 1b shows the survey spectra collected on the 0.5 M perovskite/TiO_2_/FTO/glass samples using two different excitation energies of 880 eV (SOXPES, top, red spectrum) and 2.0 keV (HAXPES, bottom, blue spectrum). As seen, when using very surface-sensitive excitation energy, we mostly see core-level emissions that originate from the top perovskite film. Nevertheless, once the excitation energy is increased to the hard X-ray range, besides the peaks related to the perovskite film, one can access the core level emissions from the buried TiO_2_ film.^39^

In our investigation, we explore the TiO_2_/perovskite interface by varying the perovskite layer thickness and employing two different photon energies. We prepared three different perovskite film thicknesses (approximately 5, 15, and 400 nm) using precursor solutions with concentrations of 0.2, 0.5, and 1.2 M, respectively, and measured them with two different photon energies. Our study is designed to achieve three key objectives: first, to scrutinize the initial interaction dynamics between the TiO_2_ substrate and the perovskite layer; second, to elucidate how reaction dynamics evolve as a function of perovskite layer thickness on TiO_2_; and ultimately, to establish a correlation between the bulk properties of perovskite and the properties at the TiO_2_/perovskite interface. This multifaceted approach provides a comprehensive understanding of the interface behavior and its implications for the overall properties of perovskite materials.

The survey spectra of bare TiO_2_ and 0.2, 0.5, and 1.2 M perovskite films on TiO_2_ measured with 2.0 and 880 eV are shown in Figure S1 of the Supporting Information. The morphology of the prepared perovskite films is investigated using top view and cross-section imaging of scanning electron microscopy (SEM) and material property by X-ray diffraction (XRD) and ultraviolet–visible (UV–vis) spectroscopy, and their photovoltaic performance is investigated in the n–i–p structure (see Figure S2, panels a and b of Figure S3, and Figure S4 of the Supporting Information, respectively). The surface morphology of the perovskite film prepared with 0.5 and 1.2 M precursor solutions shows grains in size of around 100 and 250 nm, respectively, while the thinnest film prepared with 0.2 M has the roughness of the TiO_2_ layer underneath. In the XRD pattern (see Figure S3a of the Supporting Information), we can clearly observe peaks at 14.3° and 28.5°, which correspond to the reflections originating from the perovskite structure of the films.^40^ However, it is noteworthy that the 0.2 M film exhibits considerably lower peak intensities in comparison to the other two films. This reduction in peak intensity is likely attributed to the lower thickness (∼5 nm) of the film on the TiO_2_/FTO substrate. UV–vis spectra of these film show an absorption edge at around 786.4 nm, resulting in a band gap of 1.57 eV, which is in agreement with the band gap of triple-cation-based perovskite.^39^ The PCE of the solar cells prepared from these films is around 4.7, 10.5, and 18.0%, respectively. The lower efficiency of solar cells (see Figure S4 of the Supporting Information) having 0.2 and 0.5 M perovskite films comes from the lower absorption of thin perovskite films (see Figure S3b of the Supporting Information). It is essential to highlight that the films with concentrations of 0.2, 0.5, and 1.2 M exhibit characteristics consistent with perovskite materials in terms of their morphology, crystallographic properties, optical attributes, and solar cell performance. These features make these films well-suited for further investigations that focus on interfacial studies.

**Figure 2:**
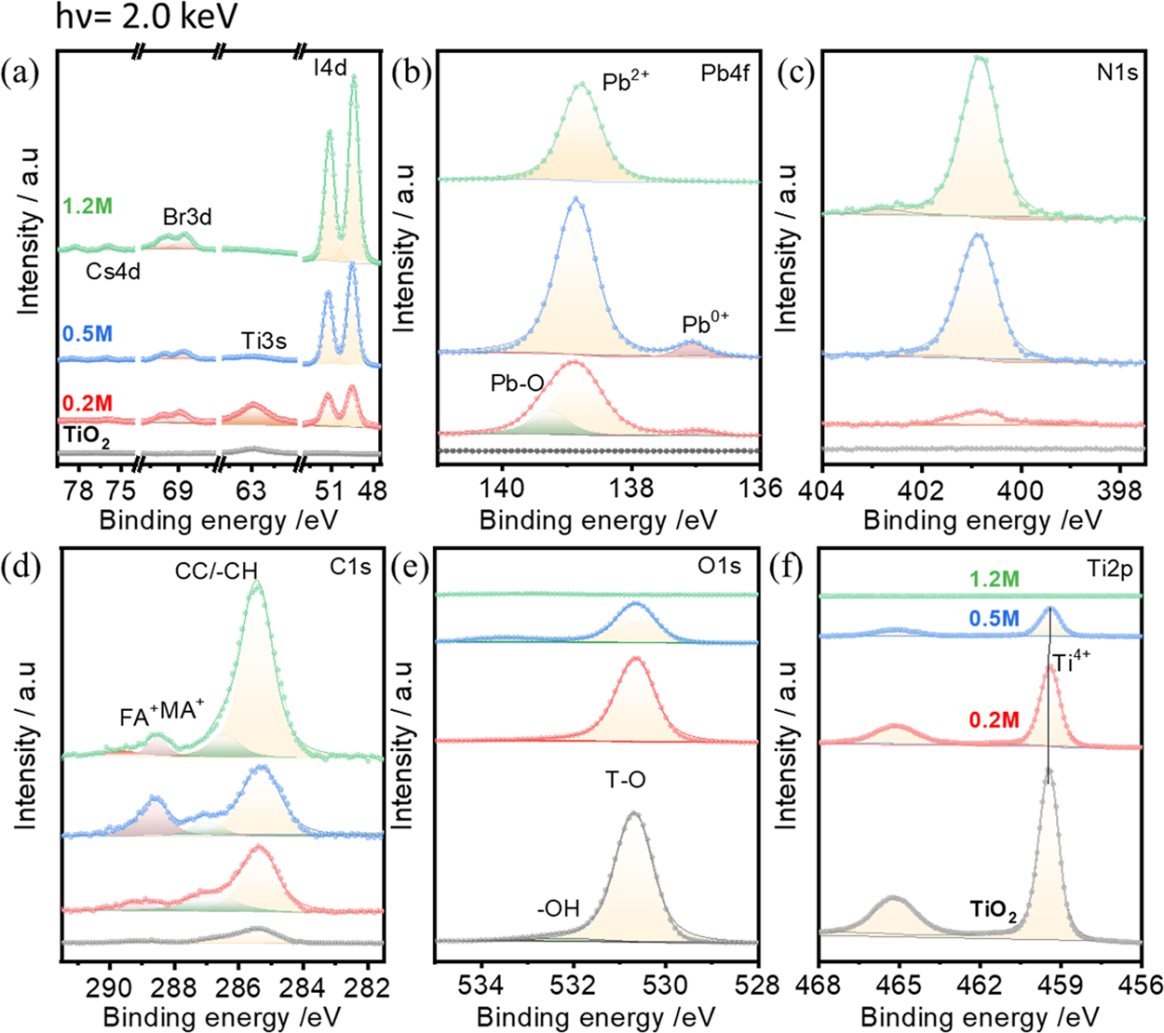
HAXPES detailed spectra of the (a) Cs 4d, Br 3d, Ti 3s, and I 4d, (b) Pb 4f_7/2_, (c) N 1s, (d) C 1s, (e) O 1s, and (f) Ti 2p core levels (including curve fit results) measured on bare TiO_2_ and 0.2, 0.5, and 1.2 M perovskite films on it using hard X-ray photoelectrons with 2.0 keV excitation energy.

To study the interface interaction between TiO_2_ and perovskite, we use the hard X-ray of photon energy of 2.0 keV for HAXPES. In Figure 2, core level spectra from HAXPES on TiO_2_ and 0.2, 0.5, and 1.2 M perovskite films are shown. The HAXPES core level spectra of Cs 4d, Br 3d, and I 4d (a), Pb 4f_7/2_ (b), N 1s (c), C 1s (d), O 1s (e), and Ti 2p (f) taken on bare TiO_2_ and with perovskite films taken using an excitation energy of the X-ray of 2.0 keV are shown in Figure 2. In Figure 2a, the binding energy in the range of 74–78, 67–71, 60–65, and 47–52 eV corresponds to the Cs 4d, Br 3d, Ti 3s, and I 4d shallow core levels. In the Pb 4f_7/2_ core level spectra (Figure 2b), one can clearly distinguish a main peak located at 138.8 eV and a low intense peak at 137.0 eV that correspond to Pb^2+^ and Pb^0^ states of lead in perovskite, respectively.^30^ The peak at 137.0 eV is clearly visible in the XPS spectra of the thin (∼5 and 15 nm) perovskites (0.2 and 0.5 M), and the Pb^2+^/Pb^0^ peak intensity ratio for these films is the same (shown in Figure S5 of the Supporting Information).

In Figure 2c, the N 1s core level spectra have a peak maximum located at 400.8 eV that is attributed to the binding energy of nitrogen in the investigated perovskite film, while in C 1s spectra (Figure 2d), three peaks, located at 285.3, 287.1, and 288.6 eV, can be seen that correspond to absorbed carbon/CH_3_I and MA^+^ and FA^+^ ions, respectively.^41,42^ In the case of a 1.2 M thick perovskite layer, a small peak at around 289.0 eV is visible, which corresponds to the absorbed −CO_3_ species. In O 1s XPS spectra (Figure 2e), the binding energy is located at 530.7 eV, which can also be assigned to the Ti–O bonding in TiO_2_.^20,43^ In Figure 2f, the Ti 2p spectra have binding energies located at 459.4 and 465.3 eV that correspond to the spin–orbit splitting of Ti 2p in TiO_2_.^10,37^ The intensities of the core level spectra related to the perovskite film, i.e., Cs 4d, Br 3d, I 4d, Pb 4f, N 1s, and C 1s, increase accordingly with the perovskite solution concentration, and at the same time, the intensities of the Ti 2p and O 1s core levels decrease, thus confirming an increase of the perovskite thickness with an increasing molar concentration of its solution.

The core level spectra of different perovskite thicknesses can further be analyzed to learn about chemical reactions occurring during the TiO_2_/perovskite interface formation. In the Pb 4f_7/2_ XPS spectra, the full width at half maximum (fwhm) value decreases with the increasing thickness of the perovskite absorber (see Figure S5 of the Supporting Information). The wider fwhm of the thinnest 0.2 M perovskite layer could be a result of the interaction of perovskite with the TiO_2_ substrate or the lower crystallinity of the perovskite film. A closer look at the binding energy position of Pb 4f shows that the Pb 4f_7/2_ spectrum for the 1.2 M film shows it to be shifted to a lower binding energy by about 0.1 eV compared to the 0.2 M film. The N 1s XPS signal for the 0.2 M sample has a shoulder at the binding energy of around 399.0 eV (see Figure S6 of the Supporting Information). This indicates that nitrogen coming from the organic cation in the perovskite solution reacts with the mesoporous TiO_2_ layer. For thicker films, i.e., 0.5 and 1.2 M, the N 1s fwhm becomes narrower and has only one component. However, after the 0.2 M perovskite layer deposition, the Ti 2p and O 1s binding energy is also shifted by 0.1 eV to a lower binding energy and stays the same for the 0.5 M sample. The origin of these shifts and shoulders as a function of perovskite thickness will be discussed in detail in the later section.

As mentioned above, the perovskite at the interface with TiO_2_ (0.2 M) has different chemical and electronic properties than that of 0.5 or 1.2 M. Therefore, to understand this interface in more detail, we use a more surface-sensitive X-ray energy of 880 eV to study the surface of TiO_2_, the interface of 0.2 M perovskite/TiO_2_, and the surface of 1.2 M perovskite.

**Figure 3:**
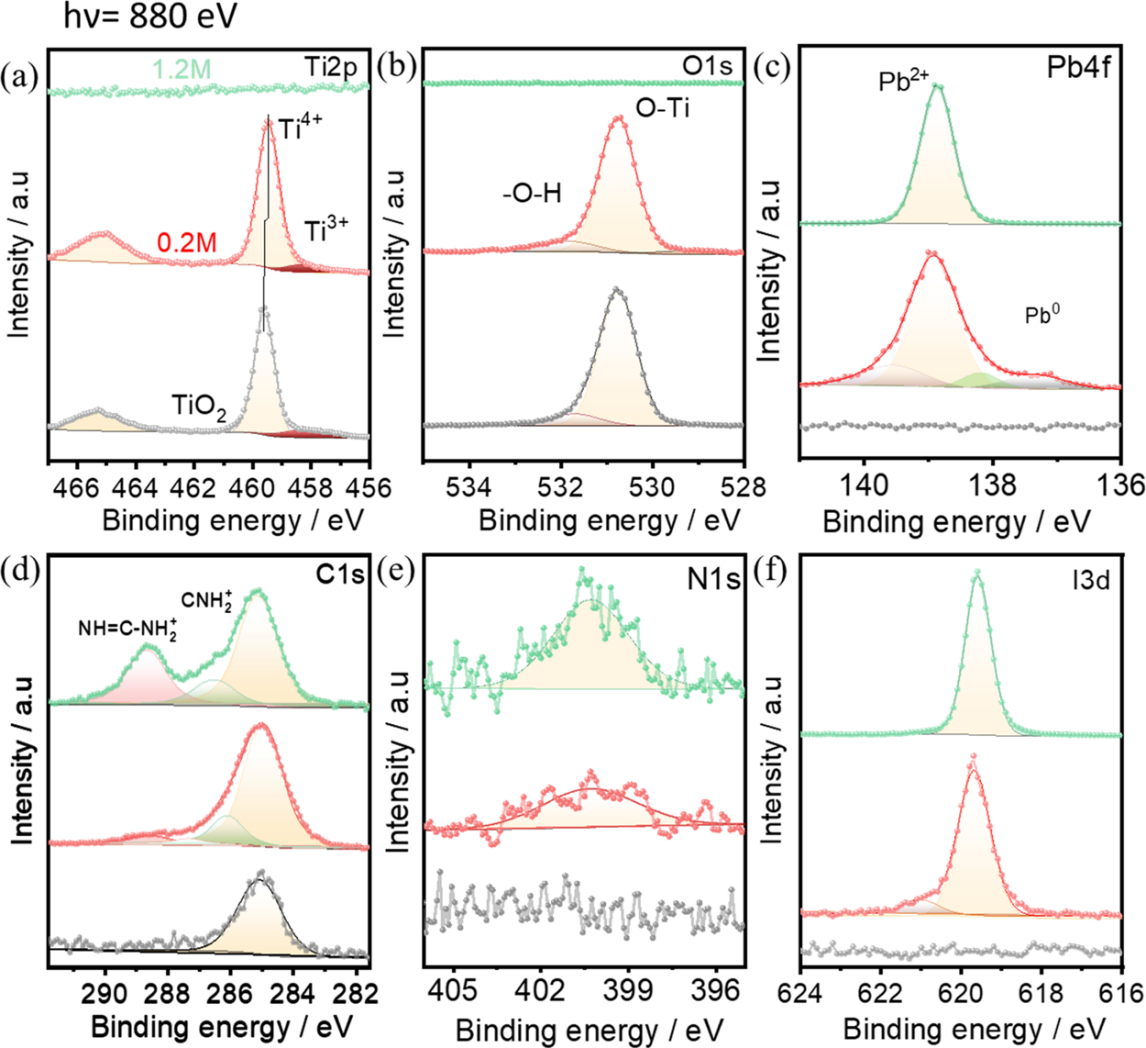
(a) Ti 2p, (b) O 1s, (c) Pb 4f_7/2_, (d) C 1s, (e) N 1s, and (f) I 3d_5/2_ core level spectra (including curve fit results) measured on the TiO_2_ substrate and 0.2 and 1.2 M perovskite layers on it collected with the soft X-ray source of energy of 880 eV.

The Ti 2p core level spectra measured with an excitation energy of 880 eV are shown in Figure 3a and indicate that the binding energy of 0.2 M perovskite shifts −0.1 eV compared to bare TiO_2_, which is also observed in the Ti 2p data measured with 2.0 keV using the HAXPES method (Figure 2f). In addition to the main peak at around 459.6 eV, a low intense peak located at the lower binding energy of 458.0 eV is visible and corresponds to the Ti^3+^ states in TiO_2_.^21,43^ Moreover, these Ti^3+^ states (oxygen vacancies) in TiO_2_ result in gap states of TiO_2_, making it more conducting.^44,45^ The relative intensity of this peak with respect to the main peak decreases after the deposition of the 0.2 M perovskite film (see Figure S7 of the Supporting Information). The decrease in intensity of Ti^3+^ states is related to the partial oxidation of Ti^3+^ to Ti^4+^ states by perovskite, which also shifts the main peak at 459.5 eV by −0.1 eV. The O 1s spectra also show a shift toward a lower binding energy by −0.1 eV, whereas a shoulder at a higher binding energy becomes more evident, which could be attributed to the −OH groups bonded to Ti or O from hydrocarbons.^20^ In the Pb 4f_7/2_ spectra (Figure 3c), four indicative peaks located at 137.1, 138.1, 138.9, and 139.6 corresponding to Pb^0^, O=Pb=O, Pb^2+^ in perovskite, and Pb–O bonds, respectively,^46^ are visible in the spectrum of the 0.2 M sample. In the case of the 1.2 M sample surface, only a single sharp peak located at 138.9 eV is visible and is attributed to the binding energy of lead in the perovskite. The presence of a considerable amount of Pb^0^ (11% of total lead) state in the 0.2 M film and its absence in the thick and bulk 1.2 M perovskite film indicate that the metallic lead state is only present at the TiO_2_/perovskite interface. Moreover, the partial oxidation (15% of Pb=O and 7% of Pb–O) of Pb observed only in the 0.2 M sample spectra indicates that a certain amount of Pb atoms from perovskite is bonded to the oxygen atoms from the TiO_2_ surface. The observed decrease in Ti^3+^ states (Figure 3a) and the presence of Pb^0^ states at the TiO_2_/perovskite interface after 0.2 M perovskite deposition indicate that perovskite oxidizes the Ti^3+^ states located at the TiO_2_ surface to Ti^4+^. This finding contradicts the theoretical assumptions that the TiO_2_/perovskite interface consists only of Pb–O–Ti bonds^47,48^ and that TiO_2_ oxidizes the perovskite layer at the interface. We experimentally found that it is not TiO_2_ that oxidizes perovskite but that the opposite happens. In other words, if all oxygen on the TiO_2_ surface would be bonded to Pb, then there will not be any visible metallic Pb^0^ states, and the intensity of the Ti^3+^ state must increase after deposition of perovskite on TiO_2_ because TiO_2_ would share oxygen with perovskite and, thus, would decrease its oxidation.

C 1s core level spectra (Figure 3d) have multiple peaks coming from −CH_3_I, MA^+^, and FA^+^ components in the perovskite film. After careful analysis of the C 1s spectra, one can see a peak located at around 287.8 eV, which can be attributed to the binding energy of C=O components in organic molecules.^49^ The reactive components (−C=O) of C 1s for 0.2 M at TiO_2_ show that the organic part of perovskite is chemically interacting with TiO_2_ at the interface. The higher photon flux at the soft X-ray beamline might have caused the surface degradation of the perovskite films, which caused the lower N 1s intensity and low signal-to-noise ratio, making the analysis challenging.

In I 3d core level spectra, a low intensity peak at a binding energy of 621.1 eV appears for the 0.2 M film, whereas it is absent for the 1.2 M perovskite film. The higher binding energy peak at the I 3d core level could originate from the interaction between I and O at the perovskite/TiO_2_ interface. This indicates that heavy elements, like Pb, take place in the interface creation between TiO_2_ and perovskite and other components, like C, from organic cation or iodine can also be bonded to the TiO_2_ underlayer.

In the theoretical studies of the TiO_2_/perovskite interface, it was shown that the oxygen vacancies responsible for defect states in TiO_2_ interact with Pb of the [PbI_6_]^−4^ octahedron in the perovskite structure.^47^ This agrees with our findings that the Ti^3+^ states (which can be understood as the measure of the oxygen vacancies in the TiO_2_ film) are oxidized at the interface with TiO_2_ after spin coating perovskite on it. The findings from HAXPES and SOXPES indicate that the interface between perovskite and TiO_2_ is not solely formed by the O–Ti–Pb bonds. Instead, all of the elements present in the perovskite crystal, except nitrogen, are chemically connected to the TiO_2_ substrate at the interface.

To understand in more detail the interfacial reaction and the defect states, we studied bare TiO_2_ and the 0.2 M perovskite/TiO_2_ samples by X-ray absorption spectroscopy (XAS) and resonance X-ray photoelectron spectroscopy.

**Figure 4:**
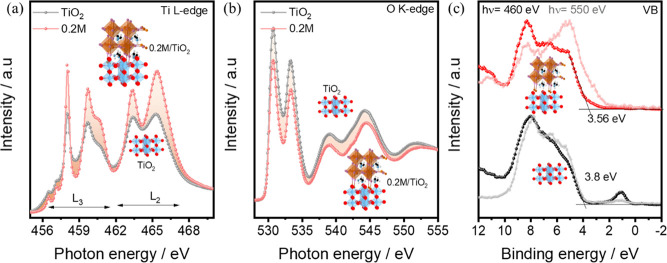
XAS spectra were collected with total electron yield mode at the (a) Ti *L*-edge and (b) O *K*-edge of TiO_2_ and 0.2 M perovskite on TiO_2_ and (c) valence band edge spectra of TiO_2_ and 0.2 M perovskite/TiO_2_ at the resonance energy of Ti 2p (460 eV) and close to the resonance energy of O 1s (550 eV).

The XAS of the Ti *L*-edge and O *K*-edge spectra of TiO_2_ (black) and 0.2 M perovskite (red) spin coated on TiO_2_ are shown in panels a and b of Figure 4, respectively. In XAS, the spectra are obtained by exciting the core electron in the occupied state to the unoccupied states of the element bonded to the other elements. The electronic transition from occupied to unoccupied states must follow the dipole selection rule. This means that, in the present case, the Ti *L*-edge and O *K*-edge electrons are excited from Ti 2p to Ti 3d and from O 1s to O 2p levels, respectively. Therefore, a change in the bonding of Ti–O can be visible in the XAS spectra of both Ti *L*-edge and O *K*-edge. The stronger the bond between the elements, the higher the unoccupied states, and hence, the higher the transition intensity visible in the XAS spectra. In the Ti *L*-edge, the energy range from 456.0 to 462.0 eV corresponds to the *L*_3_ edge, while the peaks in the range from 462.0 to 470.0 eV belong to the *L*_2_ edge. The sharp peak at 458.0 eV and the peak at 459.7 eV with a shoulder at higher photon energy are assigned to the t_2g_ and, e_g_, transitions, respectively.^50^ Similarly, these transitions are also visible in the *L*_2_ region at 463.3 eV (t_2g_) and 465.4 eV (e_g_). Bare TiO_2_ has peaks with lower intensities for both transitions at t_2g_ and e_g_ in the *L*_3_ and *L*_2_ regions than 0.2 M perovskite/TiO_2_. The lower transition intensity in bare TiO_2_ depicts that the conduction band is partially occupied.^51^ The partial occupation of the conduction band is related to the non-bonded Ti^3+^ states, which are available in bare TiO_2_ (see Figure 4a).^52^ In the literature, it has been observed that an excess of Ti^3+^ states in TiO_2_ results in a decrease in the intensity of the t_2g_ peak in XAS.^55^ In addition, the fwhm of t_2g_ at the *L*_3_ edge is higher for bare TiO_2_ than for the 0.2 M perovskites on TiO_2_. The higher fwhm at e_g_ originates typically from the deficiency in the long-range ordering of Ti–O bonds in TiO_2_.^43^ Also, the presence of the Ti^3+^ state results in the broadening of the e_g_ transition in the *L*_3_ region.^52^ Moreover, the pre-edge region in TiO_2_ has two broad continuous peaks at 456.5 and 457.1 eV, while the peaks are more dominant and discrete for 0.2 M perovskite on TiO_2_. The continuous pre-edge peak is again related to the gap states present in TiO_2_,^21,53^ which diminish after perovskite deposition.

In Figure 4b, the O *K*-edge XAS spectra show a higher absorption peak for TiO_2_ compared to the 0.2 M perovskite film spin coated on TiO_2_, quite contrary to what is observed in the Ti *L*-edge spectra. A higher intensity of the O *K*-edge in XAS corresponds to a stronger bond between Ti and O in TiO_2_. In the case of bare TiO_2_, there is only an O–Ti bond on the surface, which results in a higher intensity of t_2g_ and e_g_ in the O *K*-edge. The absorption intensity decreases considerably upon 0.2 M perovskite deposition. This infers that oxygen in the surface region of the TiO_2_ film might have been bonded to other species from the perovskite film during its spin coating. However, it is challenging to detect the resonance spectra of any other bonding species from perovskite with O as a result of a much stronger bond of Ti–O, leading to suppression of other minor signature peaks.

The O *K*-edge and Ti *L*-edge show that TiO_2_ changes its bonding nature on its surface after the deposition of 0.2 M perovskite on it. XAS also indicates that the perovskite deposition produces a decrease in the defect concentration at the interface as Ti^3+^ is converted to the Ti^4+^ states. Therefore, we measured the valence band (VB) of TiO_2_ and 0.2 M perovskite on it using two different photon energies of 460 eV (resonant to Ti 2p) and 550 eV (close to the O 1s resonance), as depicted in Figure 4c. The valence band maxima (VBMs) collected at 460 eV photon energy are found to be located at 3.8 ± 0.1 and 3.6 ± 0.1 eV for TiO_2_ and the 0.2 M perovskite layer on TiO_2_, respectively. The deep VB region spectral features for TiO_2_ and the 0.2 M perovskite layers are found to be similar. Additionally, the VBM of the perovskite layer does not match the literature value for 1.65 eV.^54^ However, a tail with a lower density of the state extended until 2.4 ± 0.05 eV could have originated from the thin perovskite layer. The discrepancy in VBM of the 0.2 M perovskite layer is coming from the fact that the perovskite layer is too thin, and second, the photon energy used here is resonating with Ti 2p, thus increasing the emission from the TiO_2_-like valence band states. PES measurements based on 460 eV excitation are very surface-sensitive, and a maximum information depth of ∼2–3 nm is expected, while at this energy, the photoelectrons selectively emitted from TiO_2_ result in the TiO_2_-like VB for the 0.2 M perovskite layer. In other words, the VB region investigated with the excitation energy close to the Ti 2p resonance results in the domination of the titanium states with a low density of states (DOS) of the perovskite film in the VB region. The VBMs of TiO_2_ and 0.2 M perovskite spin coated on TiO_2_ are located at 3.8 ± 0.1 and 1.6 ± 0.1 eV, respectively, when measured with an excitation energy of 550 eV. The photon energy of 550 eV is non-resonating with both Ti 2p and O 1s and has surface sensitivity similar to 460 eV, which is useful to identify the states in the 0.2 M perovskite layer. Therefore, with an excitation energy of 550 eV, 0.2 M perovskite has a VBM equal to the VBM of 1.2 M perovskite layers (see Figure S8 of the Supporting Information) and is similar to the reported values in the literature.^54^ Also, it can be seen that the VBM of 0.2 and 1.2 M perovskite investigated using other excitation energies (880 and 1100 eV) is located at around 1.6 ± 0.1 eV (see Figure S9 of the Supporting Information). Therefore, the VBM of perovskite investigated with the surface- and bulk-sensitive conditions is the same and agrees with the reports.

Next, using the resonant and surface-sensitive photon energies, the contribution of TiO_2_ in the VB region in 0.2 M perovskites can be distinguished, and hence, the defects at the TiO_2_/perovskite interface can be investigated. The VBs at 460 and 550 eV photon energies show a highly intense in-gap state (related to Ti^3+^) ranging from 0.4 to 1.9 eV. The intensity of the in-gap states is higher for 460 eV compared to 550 eV, showing that the defects are arising from the Ti^3+^ states in TiO_2_. In the 0.2 M perovskite layer, there are no such in-gap states visible when using 550 or 460 eV photon energy. This indicates that perovskite successfully reduced the defect states on the TiO_2_ surface. In addition, the defect states related to Ti^3+^ states decreased after perovskite deposition. The resonant VB spectra andthe Ti *L*-edge XAS results suggest that the spin coating of perovskite removes the Ti^3+^ related defect states found on the bare TiO_2_.

**Figure 5:**
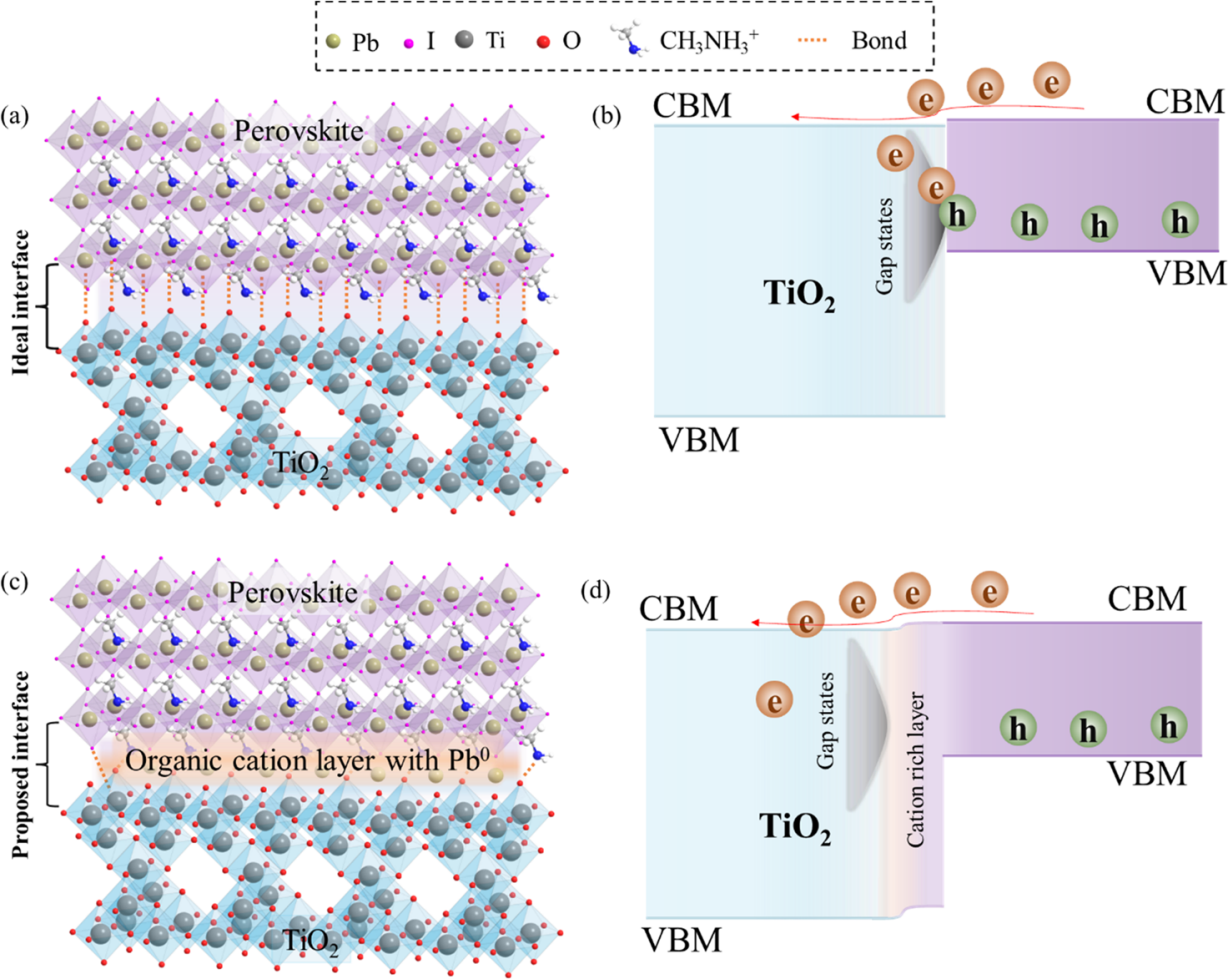
Schematic representation of the (a and b) theoretical and (c and d) experimentally verified in this work TiO_2_/spin-coated perovskite interface properties. A theoretical assumption is that (a) Pb is only attached to the Ti–O frame and (b) its consequent interface band diagram. Our experimentally verified TiO_2_/spin-coated interface shows the (c) interaction of Ti–O with Pb and organic cations and (d) consecutive band diagram.

The HAXPES and SOXPES studies of the TiO_2_/spin-coated perovskite interface show that this interface is chemically reactive during perovskite film growth on the TiO_2_ substrate. At this interface, both TiO_2_ and perovskite react favorably for electron transport from the perovskite absorber to the TiO_2_ electron transport layer (ETL). The HAXPES results show that the Ti 2p core level shifts to a lower binding energy by −0.1 eV and by the same value as Pb 4f in the 0.2 M perovskite film (Figures 2 and 3). This infers that TiO_2_ shows an upward band bending, while there is no band bending at the perovskite side as it follows the energy shift of the substrate. In addition, it is found that, at the TiO_2_/spin-coated perovskite interface, the perovskite film has a metallic state in the Pb 4f spectrum, which is not present in the 1.2 M perovskite film. On the basis of these findings, in Figure 5, we propose the schematic presentation of the theoretically driven (a and b) and experimentally determined in this work (c and d) TiO_2_/perovskite interface creation during the spin coating of the perovskite film on the mesoporous TiO_2_ film.

As indicated in the SOXPES data, the TiO_2_ film is used as an ETL in PSCs because it contains Ti^3+^ states that cause in-gap states and, hence, improve the conductivity of this high band gap (3.2 eV) material. However, as observed in Figures 4c and 5b, the in-gap states with a width of ∼1.0 eV in TiO_2_ cover the entire band gap of perovskite. In such a case, the photogenerated holes will recombine with the electrons through the in-gap states, which will lead to a drop in the performance of the solar cell devices, but this is not the case (see Figure S4 of the Supporting Information). This means that the in-gap states in the TiO_2_ layer after spin coating of perovskite and preparing devices do not interfere with interfacial charge separation. We have observed that, upon deposition of the 0.2 M perovskite on the TiO_2_ film, the Ti^3+^ states decrease and, hence, the in-gap states at the TiO_2_ surface. This decrease in Ti^3+^ states (in-gap state) does not decrease the PCE of the solar cells, which means that the in-gap states are decreased only at the TiO_2_ surface in contact with spin-coated perovskite and that the bulk properties of TiO_2_ remain intact (still in-gap states/defects are present in the TiO_2_ bulk). According to theoretical studies, perovskite attracts oxygen from the TiO_2_ layer during the spin-coating process and, thus, becomes oxidized.^55,56^ Under this circumstance, the Ti^3+^ states should increase, and there should not be any metallic lead (Pb^0^) detected in the XPS spectra. On the contrary, our experimental data collected with the advanced PES methods have revealed that the Ti^3+^ in-gap states decrease and the metallic Pb^0^ states appear at the TiO_2_/perovskite interface during spin coating of perovskite on top of the mesoporous TiO_2_ film.^56^ The interfacial reaction between TiO_2_ and perovskite during film formation can be understood through the following reactions:
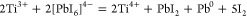
1
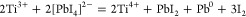
2where the [PbI_4_]^2–^ complex is created by the following reactions:
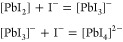
The surface oxidation of the TiO_2_ film from the Ti^3+^ to Ti^4+^ states likely occurred in conjunction with the perovskite film acting as an electron acceptor. In that case, the interaction between Ti and I is favored at the interface, which is evident in the Ti 2p spectra.^56,57^ Moreover, the higher binding energy peak of I 3d shows that a certain amount of iodine is also bonded to the surface oxygen atom. The reactive components in C 1s and N 1s of the 0.2 M perovskite layer show that there will be a reaction between the oxygen atoms on the TiO_2_ surface and the organic cations (FA^+^ and MA^+^) from perovskite. Both cations might have connected to −O–Ti through strong hydrogen bonding, resulting in C–N–H–O–Ti at the interface.^58^

If the TiO_2_/spin-coated perovskite interface would have a non-reactive nature or [PbI_6_]^4–^ bonded to O species (as shown in Figure 5a), then Ti^3+^-related in-gap states would exist at the interface, which might cause the recombination of photogenerated electron–hole pairs. The suggested band diagram for such a condition is shown in Figure 5b. Interestingly, our studies differ from the theoretical assumptions. Our experimental findings indicate that the cations and anions are actively taking part in the formation of an interface during the spin-coating process of perovskite on the TiO_2_ film. Iodine works as an electron donor, which oxidizes the Ti^3+^ states to the Ti^4+^ state and removes the interfacial defect states. The reaction of FA^+^ and MA^+^ ions with the O and I ions with Ti shows that this interface has a very thin layer composed of organic cations without any defect states, as shown in Figure 5c. The schematic band diagram for such an interface is shown in Figure 5d. The defect states are still present in the TiO_2_ film bulk after spin coating of perovskite, which causes electron transfer across the TiO_2_/spin-coated interface. On the other hand, TiO_2_ defect passivation induces Pb^0^ states, which causes an open circuit voltage loss close to 500 meV compared to the radiative limit.^59^ To maximize the performances of the PSCs, a strategy to prevent metallic Pb formation has been tried in the literature.^60,61^ For example, the additions (in low concentration) of divalent cation halide salts (e.g., SrI_2_, MgI_2_, etc.) to the precursor mixture have been shown to reduce metallic Pb formation by (i) creating a halide-rich precursor (preventing halide vacancy defects that can lead to film degradation) and (ii) allowing divalent cation incorporation into the crystal lattice, which can replace metallic Pb defects.^61^

## Conclusion

In this work, we used HAXPES, SOXPES, XAS, and resonance PES methods to investigate the TiO_2_/spin-coated perovskite interface for the first time. HAXPES and SOXPES showed that there is favorable upward band bending in the TiO_2_ film at the TiO_2_/spin-coated perovskite interface. The perovskite layer is chemically attached to the first few nanometers of the TiO_2_ film, where the interface has an organic-rich layer, resulting in surface defect passivation of TiO_2_. O from TiO_2_ is connected to organic cations (MA^+^ and FA^+^) and partly to [PbI_6_]^4–^, and Ti is attached to I from perovskite, while unreacted Pb^0^ is embedded in this organic interlayer at the interface. On the basis of the resonance PES studies of the VB edges and XAS results, we conclude that TiO_2_ at the interface with the spin-coated perovskite film is found to be less defective than that of bare TiO_2_. The perovskite film passivates the defects present on the bare TiO_2_ surface during the spin-coating process. No extra passivation intermediate layer is needed. At present, the PSCs without any interface modification show an efficiency of about 18%, which proves our finding that the interface defects that come from the TO_2_ layer are self-passivated. Nevertheless, the emergence of Pb^0^ at the interface can have detrimental consequences for device performance, resulting in a reduction in photovoltage. To mitigate the formation of Pb^0^, implementing surface modification techniques or altering the precursor chemistry represent promising strategies to enhance overall device performance. In addition, the extra interlayer may lead to the long-term stability of perovskite solar cells because TiO_2_ will not react with perovskite directly and will not catalytically degrade the device interface under UV illumination.

## Experimental Section

The perovskite solar cells are fabricated using the triple cation and double anion following the work from Saliba et al.,^39^ and the detailed fabrication part is given in the Supporting Information. In short, the FAPbI_3_ and MAPbBr_3_ solutions were mixed at a 5.7:1 (v/v) ratio. At last, a 1.5 M CsI solution in dimethyl sulfoxide (DMSO) was added to the final perovskite solution with a 5:95 (v/v) ratio to obtain the desired (Cs_5_(MA_15_FA_85_)_95_Pb(I_85_Br_15_)_3_ CsMAFA perovskite solution. For interface study and device, different concentrations, namely, 0.2, 0.5, and 1.2 M perovskite solution, were spin-coated on the TiO_2_-layered FTO substrates for three different thicknesses of perovskite films. Spiro-MeOTAD solution in chlorobenzene was spin-coated dynamically with the 4000 rpm speed to complete the device fabrication, according to the previously reported literature.^39^

The UV–vis spectroscopy of the grown film was measured with the UV–vis–near-infrared (NIR) spectrophotometer (Lambda 1300, PerkinElmer), and for the morphology of the films, field-emission scanning electron microscopy (FE-SEM) measurement was performed using the Zeiss system. The performance of the solar cells was evaluated from the *J*–*V* characteristic using a Keithley 2400 source meter and AM 1.5 G, 100 mW/cm^2^ spectrum (SINUS-70, WAVELABS).

The interface chemical and electronic properties of the TiO_2_/perovskite surface and interface were performed using soft and hard X-ray photoelectron spectroscopic methods, as shown in Figure 1. The samples were prepared and packed in N_2_-filled boxes for transportation to synchrotron centers for PES measurements. At the synchrotron beamlines, samples were unboxed and quickly inserted into the load lock of the PES system. To keep constancy and accuracy, the samples from the same batch were measured at synchrotron centers within 10 days of sample preparation. SOXPES and XAS were performed at the National Synchrotron Radiation Centre SOLARIS, Poland, at the PHELIX beamline.^62^ The X-ray of 880 eV excitation energy was incident on the sample surface, and the photoelectrons were collected onto the analyzer at a takeoff angle of 90°. The depth of information at this photon energy is around ∼8 nm; i.e., the photoelectrons measured here are from the surface to a maximum depth of 8 nm of the film.^63^ XAS at the O *K*-edge and T *L*-edge were measured using the total electron yield method. The HAXPES measurements were conducted at the HIKE located at BESSY II KMC-1 beamline at HZB.^64,65^ Two excitation energies of 2.0 and 5.0 keV of the X-ray were used for higher depth information on the perovskite films. The X-ray was incident at a gracing angle on the sample surface to have a photoelectron takeoff angle close to 90°. The information depth for 2.0 is about ∼18.^66^ However, the exact information depth of perovskite films at these photon energies is unknown, considering the organic–inorganic hybrid nature of this material.

It is widely acknowledged that perovskite materials can undergo chemical transformations when exposed to X-ray irradiation, which can potentially impact the accuracy and interpretation of experimental results. To mitigate this possibility, we adopted a strategy of deliberately defocusing the high-intensity soft X-ray beam, thereby minimizing the potential for beam-induced damage. We also minimize the measurement time by acquiring each spectrum within a short time frame of 1–2 min. In contrast, when working with hard X-rays, the intensity was relatively low, and as a result, we observed no discernible degradation of the perovskite material.

The spectra collected at both synchrotron facilities were calibrated with respect to the Au 4f core level spectra measured at each beamline. For analysis, the spectra are fitted with the Casa XPS software using the Shirley background and a fwhm of 1.2 ± 0.3 eV. The quantification for Pb and Ti species was performed using the area under the curve. In Table S1 of the Supporting Information, we present the binding energy values for various core levels measured using two different photon energies. It is important to note that, as a result of variations between sources and instruments, the error in determining binding energy can be approximately ±0.1 eV. Additionally, we provide information on the possible chemical structures associated with each core level for reference. This table serves as a valuable reference for understanding the core levels and their associated binding energies, considering the potential measurement error.

## Supporting Information Available

Survey spectra of TiO_2_ and 0.2, 0.5, and 1.2 M perovskite films at an excitation energy of 0.88 and 2.0 keV (Figure S1), surface morphology of perovskite films prepared in the upper panel and cross section with 0.2, 0.5, and 1.2 M concentrations of the perovskite precursor (Figure S2), XRD pattern of FTO, TiO_2_/FTO, and 0.2, 0.5, and 1.2 M concentrations of the perovskite precursor coated on TiO_2_/FTO and absorption spectra of 0.2, 0.5, and 1.2 M perovskite films measured by UV–vis spectroscopy (Figure S3), current density–voltage (*J*–*V*) characteristics of solar cells prepared with 0.2, 0.5, and 1.2 M perovskite solution and statistics of the number of devices for films prepared with each concentration (Figure S4), Pb 4f_7/2_ spectra measured with an excitation energy of 2.0 keV for 0.2, 0.5, and 1.2 M perovskite films and intensity normalized to the highest intensity (Figure S5), N 1s spectra perovskite film deposited with 0.2 M solution concentration measured with the excitation energy of 880 eV (Figure S6), normalized intensity of Ti 2p spectra of TiO_2_ and 0.2 M perovskite on TiO_2_ measured using a photon energy of 880 eV and TiO_2_ and 0.2 and 0.5 M perovskite samples with 2.0 keV photon energy (Figure S7), valence band maxima of TiO_2_ and perovskite film prepared with 0.2 and 1.2 M precursor solutions measured using a photon energy of 460 eV (Figure S8), valence band maxima of TiO_2_ and perovskite film prepared with 0.2 and 1.2 M precursor solutions measured using photon energies of 1100 and 880 eV (Figure S9), binding energy of core level spectra for prominent peaks at two photon energies and chemical nature of each peak (Table S1), and device fabrication (PDF)
