# Supplementary material for: Unraveling the Role of Perovskite in Buried Interface Passivation
Source: ACS Appl Mater Interfaces. 2023 Nov 22;15(48):56500–10. doi: 10.1021/acsami.3c13085 (PMC10711719; doi:10.1021/acsami.3c13085)
Supplement: Supplementary file 1 — am3c13085_si_001.pdf [file am3c13085_si_001.pdf]

Supporting Information

Unraveling the Role of Perovskite in Buried Interface Passivation

*Chittaranjan Das *, Rajarshi Roy, Mayank Kedia , Małgorzata Kot , Weiwei Zuo , Roberto Félix, Tomasz Sobol , Jan Ingo Flege , and Michael Saliba **

Corresponding Author: (chittaranjan.das@ipv.uni-stuttgart.de, michael.saliba@ipv.uni-stuttgart.de)

Chittaranjan Das, Rajarshi Roy, Mayank Kedia, Weiwei Zuo, and Michael Saliba

Institute for Photovoltaics (ipv), University of Stuttgart, Pfaffenwaldring 47, 70569 Stuttgart, Germany

Chittaranjan Das, Mayank Kedia and Michael Saliba

Helmholtz Young Investigator Group, IEK5-Photovoltaik, Forschungszentrum Jülich, 52425 Jülich, Germany

Małgorzata Kot and Jan Ingo Flege

Chair of Applied Physics and Semiconductor Spectroscopy, Brandenburg University of Technology Cottbus-Senftenberg, Konrad-Zuse-Straße 1, 03046 Cottbus, Germany

Roberto Félix

Department Interface Design, Helmholtz-Zentrum Berlin für Materialien und Energie GmbH (HZB), Hahn-Meitner-Platz 1, 14109 Berlin

Tomasz Sobol

SOLARIS National Synchrotron Radiation Centre, Jagiellonian University, Krakow, Poland

Author Contributions: (Chittaranjan Das and Rajarshi Roy are equal co-authors)

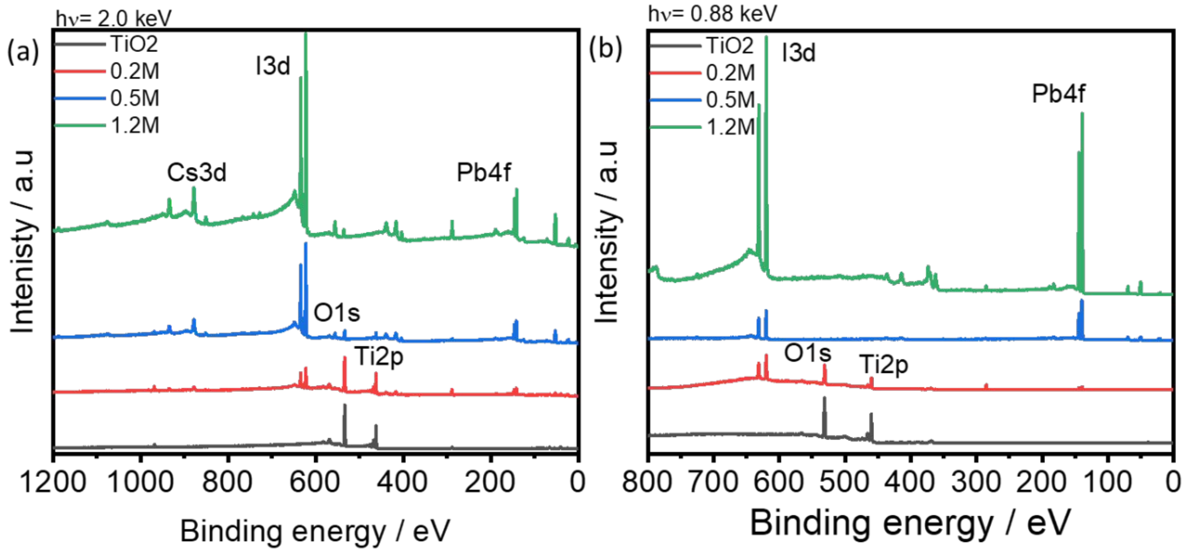

Figure S1: *Survey spectra of TiO_2, 0.2 M. 0.5 M and 1.2 M perovskite film at an excitation energy of 0.88 keV and 2.0 keV in figure (a) and (b), respectively.*

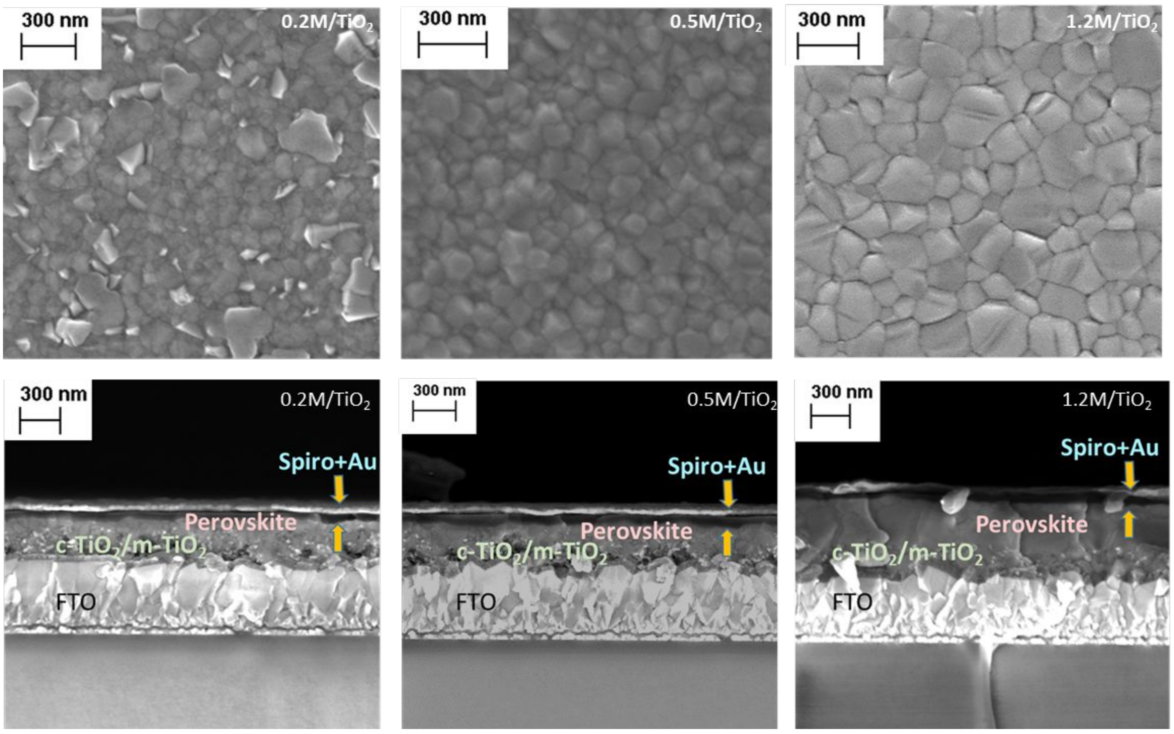

Figure S2. *The surface morphology of perovskite films prepared in the upper panel and the cross-section with 0.2, 0.5, and 1.2 M concentrations of perovskite precursor.*

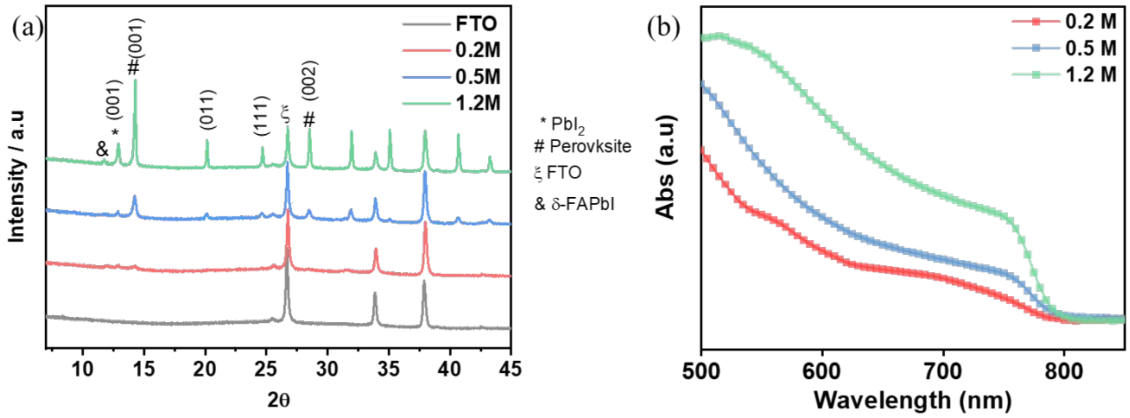

Figure S3. *The X-ray diffraction pattern of FTO, TiO_2/FTO and 0.2, 0.5, and 1.2 M concentrations of perovskite precursor coated on TiO_2/FTO (a). The absorption spectra of 0.2, 0.5, and 1.2 M perovskite film measured by UV-vis spectroscopy.*

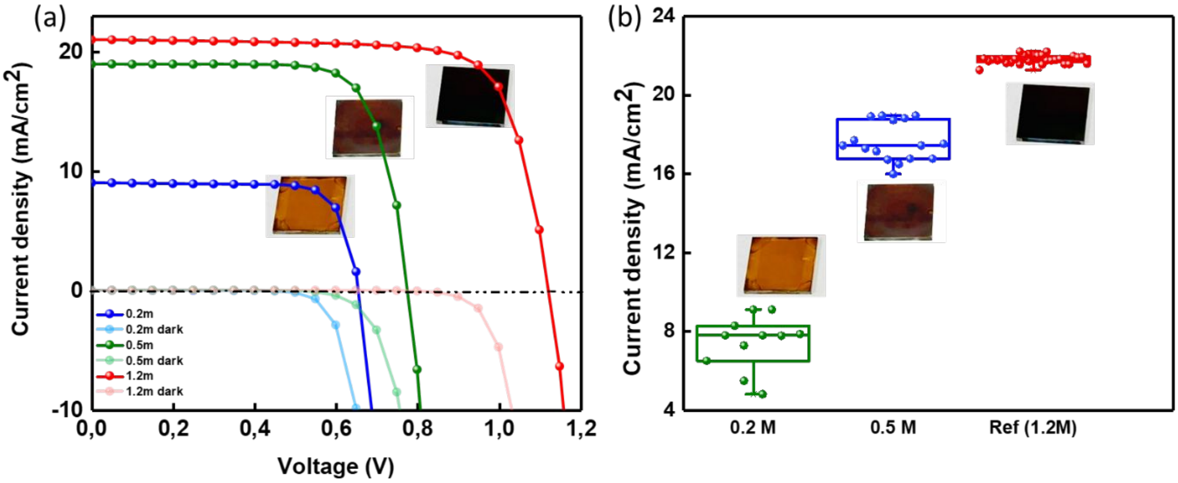

Figure S4. *Current density-voltage (J-V) characteristics of solar cells prepared with 0.2, 0.5, and 1.2 M perovskite solution (a) and the statistics of the number of devices for films prepared with each concentration (b).*

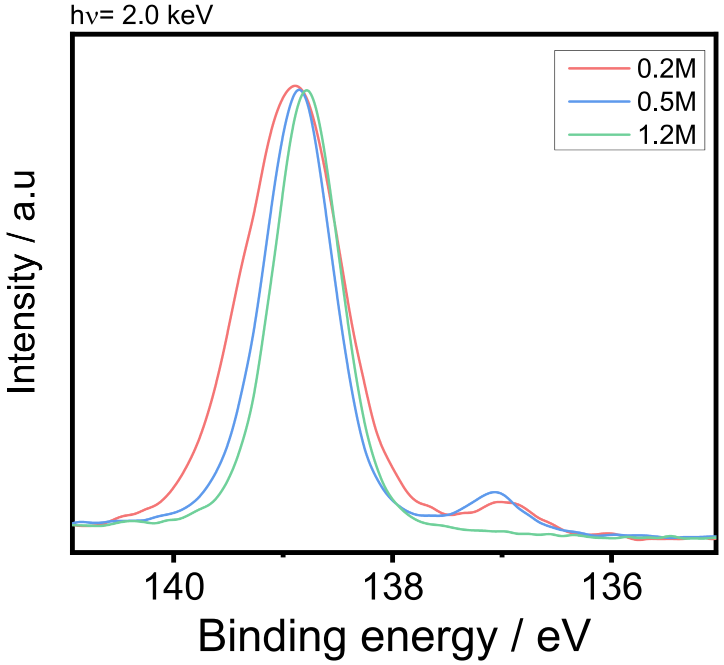

Figure S5: *The Pb4f$_{7/2}$ spectra measured with excitation energy of 2.0 keV for 0.2, 0.5 and 1.2 M perovskite films and the intensity is normalized to the highest intensity.*

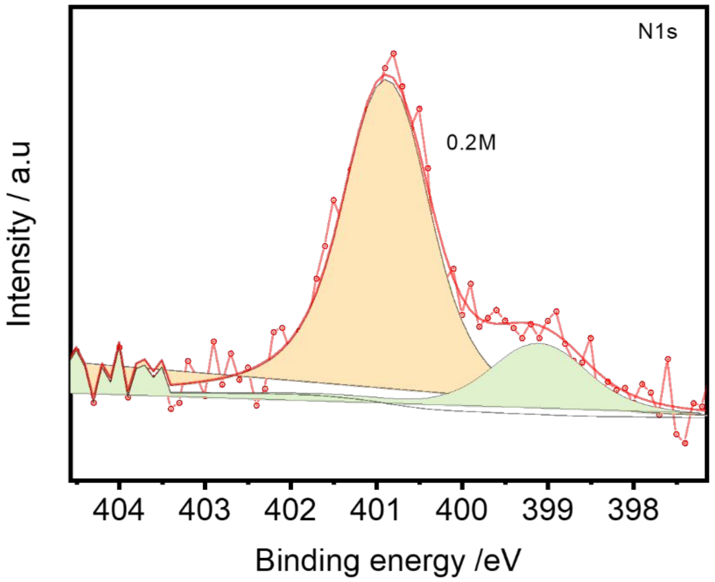

Figure S6: *The N1s spectra perovskite film deposited with 0.2 M solution concentration measured with the excitation energy of 880 eV.*

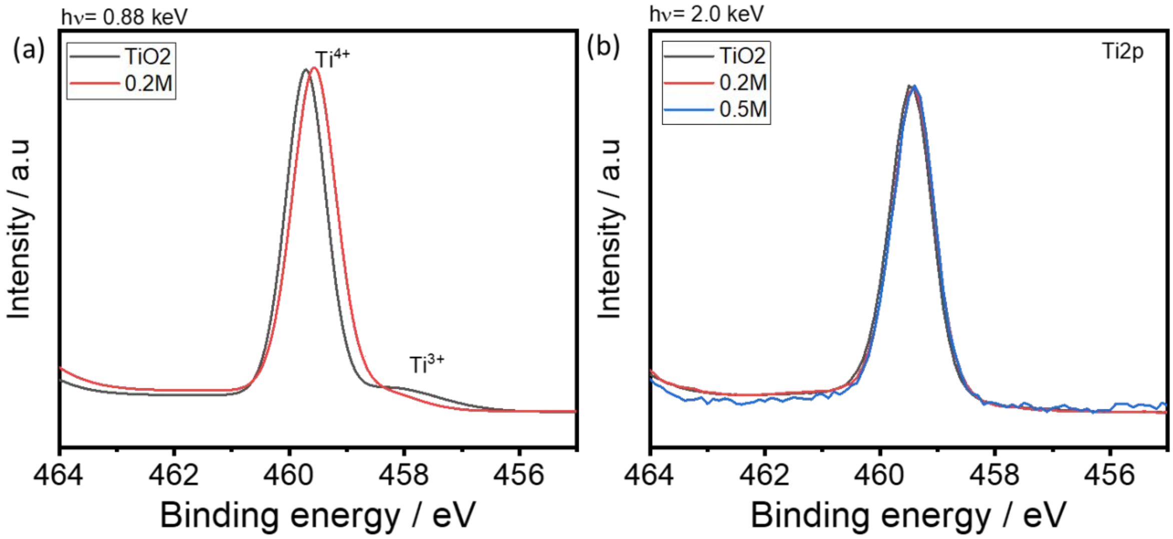

Figure S7. *The normalized intensity of Ti2p spectra of* TiO_2 *, 0.2 M perovskite on* TiO_2 *measured using a photon energy of 880 eV (a) and for* TiO_2*, 0.2 M and 0.5 M perovskite samples with 2.0 keV photon energy (b). The* Ti^{3+} *intensity at the binding energy around 458 eV decreased after the deposition of 0.2 M perovskite.*

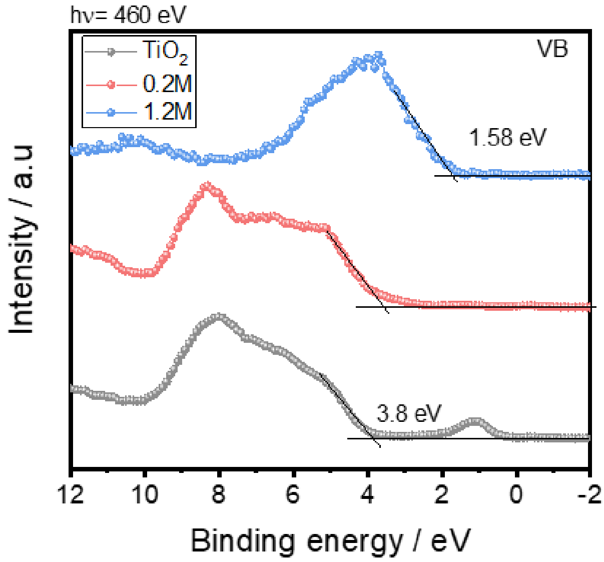

Figure S8. *Valence band maxima of* TiO_2*, perovskite film, prepared with 0.2 M and 1.2 M precursor solution, measured using a photon energy of 460 eV.*

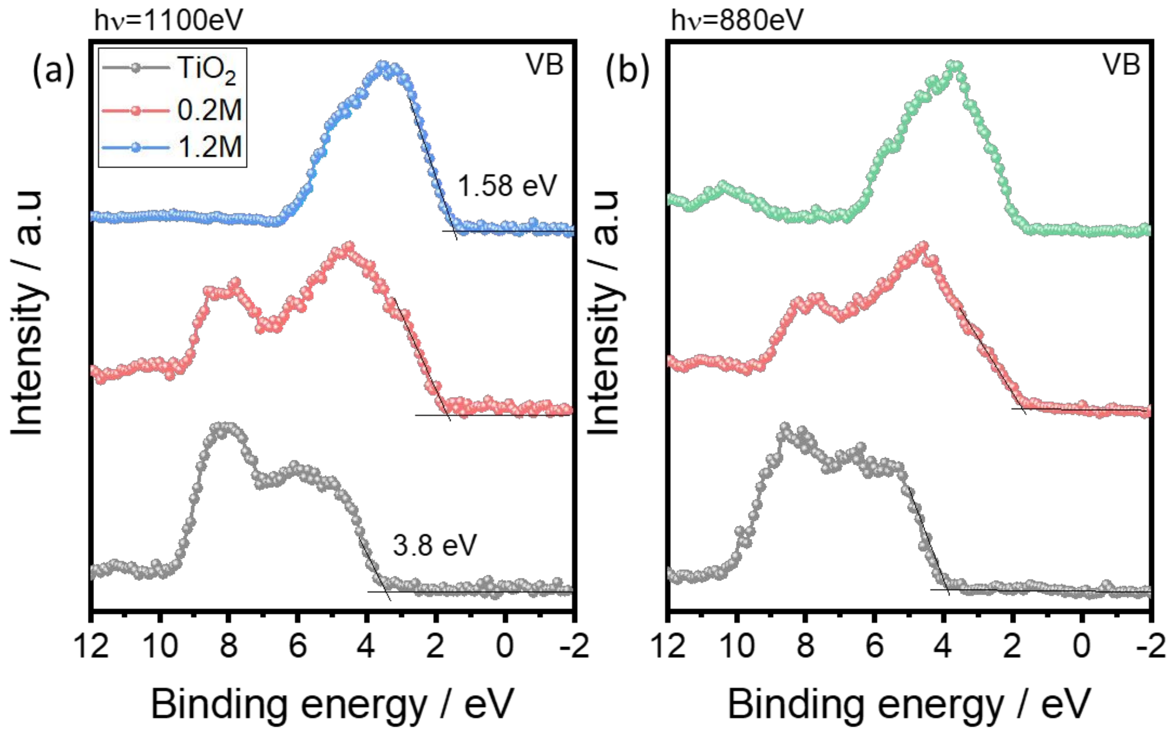

Figure S9. *Valence band maxima of TiO_2, perovskite film prepared with 0.2 M and 1.2 M precursor solution measured using photon energies of 1100 eV (a) and 880 eV(b).*

Table S1. The binding energy of core level spectra for prominent peaks at two photon energies and the chemical nature of each peak.

Core level	Photon energy / eV	
	880	2000
I4d		49.4±0.1(Perovskite)
Br3d		68.6±0.1(Perovskite)
Cs4d		75.9±0.1(Perovskite)
Pb4f	137.1±0.1 (Pb^0)<br>138.1±0.1 (O=Pb=O)<br>138.8±0.1 (perovskite)<br>139.6±0.1 ($Pb-CO_3$)	137.1±0.1 (Pb^0)<br>138.8±0.1(Perovskite)<br>139.3±0.1 (Pb-O)
C1s	285.1±0.1(C-C /-CH)<br>286.6±0.1(MA),<br>288.6±0.1(FA)<br>287.8 (-C=O)	285.4±0.1(C-C /-CH)<br>286.9±0.1(MA)<br>288.6±0.1(FA)<br>289.0±0.1($-CO_3$ absorbed)
N1s	399.5±0.1(Perovskite+ X-ray damaged)	400.7±0.1(Perovskite)
Ti2p	459.7±0.1(TiO_2)	459.5±0.1(TiO_2)
O1s	531.0±0.1(O-Ti)<br>532.0±0.1(-OH)	530.6±0.1(O-Ti)<br>533+±0.1(-OH)
I3d	619.6 (Perovskite)<br>621.1 (I-O)	

Device Fabrication:

Fluorine-doped tin oxide (FTO) substrates with a sheet resistance of 9 Ω /sq were thoroughly cleaned with 2% Hellmanex water solution, deionized water, acetone and isopropanol in an ultrasonic bath for 15 mins respectively. Then the washed substrates were treated with the UV-Ozone cleaner (Oscilla) for 15 mins for each step.

A thickness of 30 nm of TiO_2 compact layer was deposited on the cleaned FTO substrates with the spray pyrolysis method at 450 °C from a precursor solution of titanium diisopropoxide bis(acetylacetonate) in anhydrous ethanol. Once the spraying is done, the substrates were kept at 450 °C for another 45 mins and cooled down at room temperature eventually. Then a mesoporous TiO_2 layer was deposited on the sprayed substrates by spin coating for 10s at 4000 rpm with an acceleration of 1000 rpm s^{-1}. A 30 nm particle paste (GreatCell Solar) diluted in ethanol was used as the precursor solution to achieve 150-200 nm of mesoporous TiO_2 layer. Once the spin coating finished, the substrates were heated for 10 mins at 100 °C followed by annealed at 450 °C for 30 mins under a dry air flow [1].

The perovskite solution of $(Cs_5(MA_{15}FA_{85})_{95}Pb(I_{85}Br_{15})_3$ (CsMAFA) was prepared from a previously reported literature [2]. Initially $PbBr_2$ and PbI_2 solutions with 1.5 M nominal concentration were prepared in 4:1 (v:v) mixture of DMF:DMSO and were heated at 60 °C overnight. The Pb-based stock solutions were added to organic precursors such as MABr and FAI to obtain the final 1.24 M perovskite precursor solution. The $FAPbI_3$ and $MAPbBr_3$ solutions were mixed in at 5.7:1 (v:v) ratio. At last, a 1.5 M CsI solution in DMSO was added to the final perovskite solution with a 5:95 (v:v) ratio to obtain the desired CsMAFA perovskite solution. The perovskite solution was then spin coated on the TiO_2-layered FTO substrates with 5s of acceleration to 4000 rpm followed by 35s steady rotation at 3500 rpm then annealed at 100 °C for 30 mins to obtain 400-500 nm thick perovskite layer [3]. The perovskite deposition was performed in the glovebox to avoid humidity.

Once the substrates were annealed they were cooled down at room temperature and S*piro-OMeTAD* solution in chlorobenzene was spin coated dynamically with the 4000 rpm speed according to the previously reported literature [4,5].

At last, 100 nm of gold was deposited as the counter electrode under high vacuum.

REF:

1) Turren-Cruz, S.-H., Hagfeldt, A. and Saliba, M. (2018) "Methylammonium-free, high-performance, and stable perovskite solar cells on a planar architecture," Science, 362(6413), pp. 449–453.

2) Al-Ashouri, A. et al. (2019) “Conformal monolayer contacts with lossless interfaces for perovskite single junction and Monolithic Tandem Solar Cells,” Energy & Environmental Science, 12(11), pp. 3356–3369.

3) Köbler, H. et al. (2021) “The challenge of designing accelerated indoor tests to predict the outdoor lifetime of perovskite solar cells.”

4) Saliba, M. et al. (2016) “Incorporation of rubidium cations into perovskite solar cells improves photovoltaic performance,” Science, 354(6309), pp. 206–209.

5) Saliba, M. et al. (2016) “Cesium-containing triple cation perovskite solar cells: Improved stability, reproducibility and high efficiency,” Energy & Environmental Science, 9(6), pp. 1989–1997.
